# Integrating demography and distribution modeling for the iconic *Leontopodium alpinum* Colm. in the Romanian Carpathians

**DOI:** 10.1002/ece3.7864

**Published:** 2021-08-25

**Authors:** Lăcrămioara M. Maghiar, Ilie A. Stoica, Andrew J. Tanentzap

**Affiliations:** ^1^ Institute of Biological Research Branch of the National Institute of Research and Development for Biological Sciences Cluj‐Napoca Romania; ^2^ Ecosystems and Global Change Group Department of Plant Sciences University of Cambridge Cambridge UK

**Keywords:** biotic interactions, climate change, global change ecology, human exploitation, long‐term population persistence, plant fitness, species distribution models

## Abstract

1. Both climate change and human exploitation are major threats to plant life in mountain environments. One species that may be particularly sensitive to both of these stressors is the iconic alpine flower edelweiss (*Leontopodium alpinu*m Colm.). Its populations have declined across Europe due to over‐collection for its highly prized flowers. Edelweiss is still subject to harvesting across the Romanian Carpathians, but no study has measured to what extent populations are vulnerable to anthropogenic change.
2. Here, we estimated the effects of climate and human disturbance on the fitness of edelweiss. We combined demographic measurements with predictions of future range distribution under climate change to assess the viability of populations across Romania.
3. We found that per capita and per‐area seed number and seed mass were similarly promoted by both favorable environmental conditions, represented by rugged landscapes with relatively cold winters and wet summers, and reduced exposure to harvesting, represented by the distance of plants from hiking trails. Modeling these responses under future climate scenarios suggested a slight increase in per‐area fitness. However, we found plant ranges contracted by between 14% and 35% by 2050, with plants pushed into high elevation sites.
4. *Synthesis*. Both total seed number and seed mass are expected to decline across Romania despite individual edelweiss fitness benefiting from a warmer and wetter climate. More generally, our approach of coupling species distribution models with demographic measurements may better inform conservation strategies of ways to protect alpine life in a changing world.

## Graphical abstract

Few studies have compared how different anthropogenic stressors impact alpine plant life. We do so for an iconic European plant by combining species distribution models with demographic data, heeding growing calls for this integration. We find population persistence is at high risk of extinction across a massively understudied geographic area.

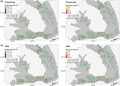

## 1 INTRODUCTION

Climate change threatens many alpine plants, especially those with relatively narrow environmental niches (Gottfried et al., 2012; Pauli et al., 2012). At the European level, warm‐adapted species have been found to expand their range to higher altitudes to the detriment of cold‐adapted neighbors (Gottfried et al., 2012). Consequently, both the composition and structure of high‐mountain plant communities are changing across several European countries (Erschbamer et al., 2011; Evangelista et al., 2016; Fernández Calzado et al., 2012; Pauli et al., 2007). Many studies have also observed an increase in species richness at high altitudes in several mountain ranges, likely increasing interspecific competition (Britton et al., 2009; Erschbamer et al., 2011; Holzinger et al., 2008; Matteodo et al., 2013; Pauli et al., 2007).

Adding to the impacts of a changing climate, human disturbance is also a major threat to plant life in populated mountain ranges, such as those found across Europe. Many mountain plants are harvested by humans, such as for food, medicinal, ornamental, and cultural purposes (Allen et al., 2014; Hinsley et al., 2018). For example, the use of *Gentiana lutea* roots in traditional medicine has led to this species being classed as endangered across parts of Europe (Catorci et al., 2014). Similarly, harvesting for medicinal purposes, predation by herbivores, and low genetic variability among populations of *Artemisia granatensis*, an endangered alpine species endemic to the Sierra Nevadas in Spain, have decreased the number of mature adults of this species by more than 50% between 1994 and 2003 (Hernández‐Bermejo et al., 2011). In all cases, long‐term population persistence will depend on what plant tissues are lost and how plant reproductive strategies respond to disturbance. Removal of flowers may be particularly harmful for the persistence of plants that only reproduce once in their lifetime and die thereafter, that is, those that are monocarpic (Law & Salick, 2005). Polycarpic or clonal species may in contrast compensate for flower loss by reallocating resources into remaining seeds or future reproduction (Ehrlén & Van Groenendael, 2001; Gómez & Fuentes, 2001; Olejniczak, 2011). For example, Lehtilä and Ehrlén (2005) found that experimental flower removal from *Primula veris* in its late flowering stage increased seed size by 33% compared with the control plants.

Species distribution models (SDMs) can help predict how climate change and other human‐induced threats will impact the future range dynamics of alpine plants (Bakkenes et al., 2002; Casalegno et al., 2010; Thuiller et al., 2005), but these approaches rarely consider the capacity of populations to regenerate and persist in space. SDMs typically focus on delineating the potential distribution of species based on associations between species occurrences and environmental conditions, mostly climate (Guisan & Zimmermann, 2000). Few studies have included human disturbance alongside climate projections, despite the former being a more immediate and larger cause of extinction (Maxwell et al., 2016). In one study that did, Pearson et al., (2004) found that the availability of suitable land cover restricted the range of *Erica tetralix* in Britain within otherwise climatically suitable habitat. Even accounting for conditions other than climate, different fecundity or survival rates across populations of a species might generate different responses to environmental change (Swab et al., 2015). Thus, SDMs can produce biased estimates of future extinction risk if they only consider environmental tolerances and omit measures relating to fitness. In the European Alps, habitats were identified as suitable for occupancy of alpine plant species under future climate change despite dispersal and demography being poorly adapted to the new conditions in some sites (Dullinger et al., 2012). Ultimately, combining SDMs with demographic information can improve our understanding of the relative importance of climate versus other human‐induced threats to the persistence of alpine plant species.

Here, we focus on identifying the relative importance of climate and human disturbance for the persistence of edelweiss (*Leontopodium alpinum*, Asteraceae), one of the most iconic flowers of Europe's mountains. We surveyed both seed mass and seed number at seven sites along a 275 km transect across Romania. Both seed mass and number are fitness‐related traits that are important for ensuring long‐term population persistence (Adler et al., 2014; Cochrane et al., 2015). Although short‐term population viability may also depend on other vital rates, such as adult survival and growth (Keller & Vittoz, 2015), sexual reproduction can enhance the ability of plants to track future climate change by promoting genetic variation, dispersal, and colonization, especially compared with vegetative organs (Weppler et al., 2006). Higher seed number may connect naturally fragmented, isolated, and small populations of edelweiss while larger seeds may withstand better environmental stressors such as deep shade or drought (Bruun & Ten Brink, 2008). We consequently predicted that climate change would reduce the probability of occurrence of edelweiss across Romania and that both fitness measures would decrease with climate change and human disturbance, thereby threatening future population persistence. Our work builds upon an emerging consensus for the need to link projections of future species distributions with key demographic processes to forecast better the potential impact of global change on species distributions (Fordham et al., 2013; Normand et al., 2014; Swab et al., 2015).

## 2 MATERIALS AND METHODS

### 2.1 Study species

Edelweiss is a small (ca. 10–50 cm tall) herbaceous perennial with a widespread disjunct distribution across Eurasia. The plant reproduces both clonally by rhizomes and sexually by seeds when rosettes have accumulated sufficient resources for flowering (Keller & Vittoz, 2015). After reproduction, the apical meristem dies, but clonal growth ensures genets can persist and produce new ramets and eventual flowers (Keller & Vittoz, 2015).

Over‐collection of edelweiss from its natural habitat led to early conservation measures in European countries such as Switzerland and Austria, where it has been protected since 1878 and 1887, respectively (Pop, 1939). Although edelweiss is listed as being of least concern across the whole of Europe according to the IUCN Red List (Khela, 2013), it is critically endangered in Albania (Red List of Wild Flora & Fauna Albania, 2013), regionally endangered in Austria (Niklfeld & Schratt‐Ehrendorfer, 1999), endangered in Bulgaria (Petrova et al., 2009), endangered in Germany (Bundesamt für Naturschutz, 2012), near threatened in Slovakia (Eliáš et al., 2015), least concern in Switzerland but listed as critically endangered and near threatened in some regions (Moser et al., 2002), and critically endangered in Ukraine (Chorney & Kyyak, 2009). In the Carpathian List of Endangered Species, edelweiss is listed as vulnerable (Tasenkevich, 2003). The species is protected in Romania and included as vulnerable and rare on the Romanian Red List of Vascular Plants (Oltean et al., 1994). Currently, there has not been any national‐level assessment of edelweiss in Romania despite the potentially large area of available habitat in the relatively sparsely populated Carpathians, with studies undertaken at a local level highlighting its vulnerability to harvesting (Oprea & Sîrbu, 2008).

### 2.2 Study sites and seed collection

**FIGURE 1:**
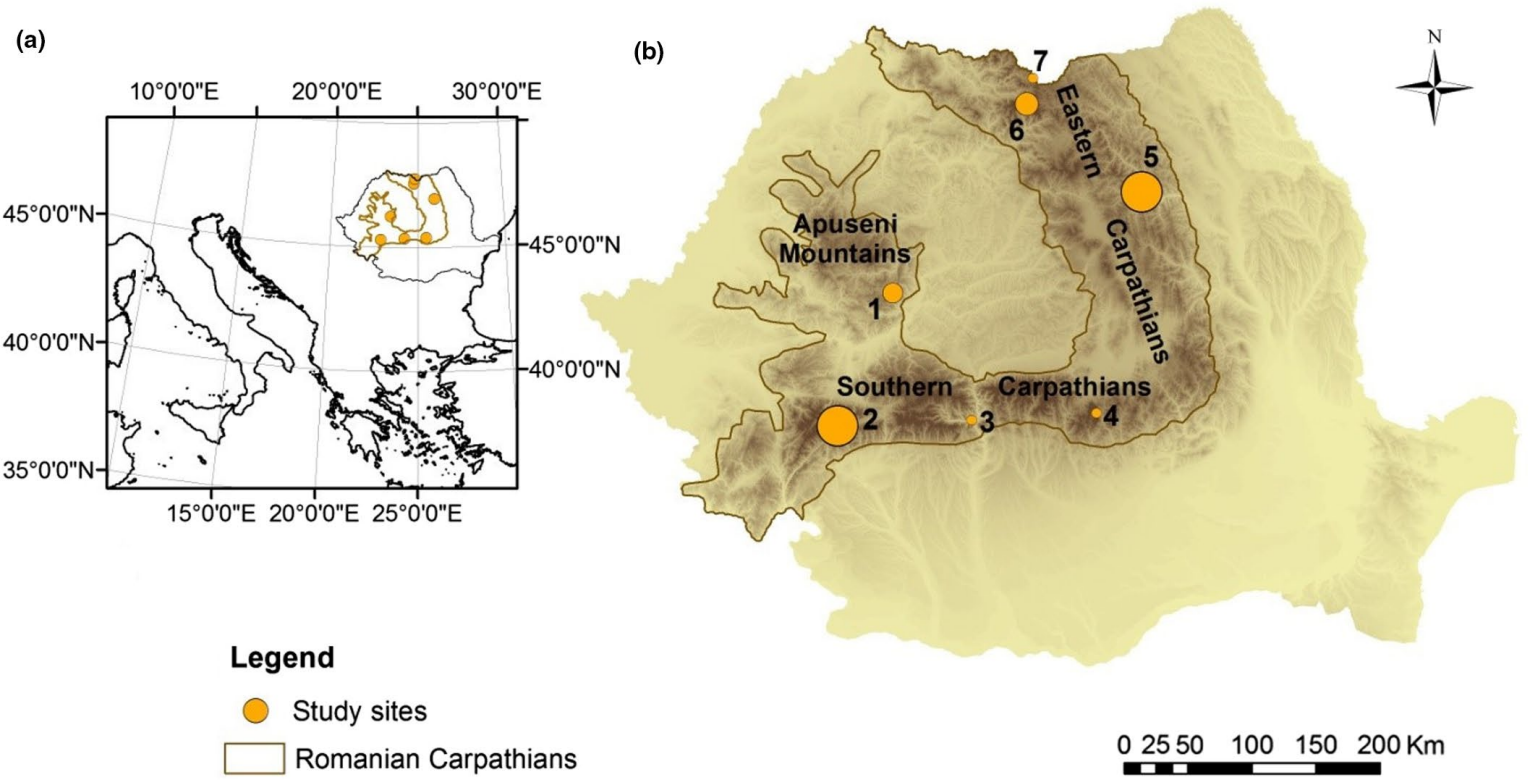
Location of study area in Europe (a) and study sites within the study area (b): 1—Întregalde Gorges; 2—Piule‐Iorgovanu Mountains; 3—Doabra Valley; 4—Bucegi Mountains; 5—Ceahlău Mountains; 6—Rodna Mountains; 7—Coman Valley. The size of the symbols in panel b is proportional to the number of plots. *N* = 3–13 plots per site

We established 45 1 m × 1 m plots in areas with known edelweiss populations across the Carpathian Mountains, Romania. The plots were nested within seven massifs with altitudes ranging between 394 m a.s.l. (Doabra Valley–Lotru Mountains) and 2,286 m a.s.l. (Bucegi Mountains) and in areas with varying levels of disturbance (Figure 1). Human disturbance was estimated for each plot as the distance to the nearest marked hiking trail. Hiking trails provide accessibility to edelweiss and should elevate the risk of harvesting immediately along the trail. The impact of human disturbance should therefore decrease with increasing distance from trails (Huang et al., 2015; Kutiel et al., 1999). Approximately 753 marked hiking trails were used for calculating distances. Trails were digitized from airborne imagery and obtained as KMZ, KML, or GPX files from the Romanian Mountain Rescue websites, websites of natural and national parks, directly from the administration of natural and national parks, and two popular enthusiast websites: http://muntii‐nostri.ro/ and https://gis.modulo.ro/.

Between August and September 2015, when the achene fruits of edelweiss were ripe, we surveyed seed production. To minimize our impacts on local populations, we subsampled diaspores (hereafter called seeds) at the level of individual flowering heads (i.e., anthodiums). In each plot, we collected five anthodiums from each of three randomly chosen inflorescences and recorded the heights of the inflorescences. Seed number per inflorescence was derived by multiplying the average number of seeds per anthodium by counts of all the anthodiums in an inflorescence. We could then multiply the average seed number per inflorescence by the total number of inflorescences to scale seed production to the plot level. Similarly, seed mass per inflorescence was derived by multiplying the average fresh seed mass per anthodium by anthodium counts per inflorescence and then converted to dry masses using the formula: dry mass = 0.89 × fresh mass (*R* ^2^ = 0.97, *n* = 37). We could then multiply the average dry seed mass per inflorescence by the total number of inflorescences to scale seed production to the plot level.

In each plot, we also recorded the ground cover of (semi‐)woody plants, hemiparasites, and cushion plants (Table S1). These three functional groups can have both positive and negative impacts on alpine plant communities (Antonsson et al., 2009; Gabay et al., 2012; Kröpfl et al., 2002; Liczner & Lortie, 2014; Spasojevic & Suding, 2011), and so could contribute to variation in seed production among plots. We estimated the abundance of these groups from traditional Braun‐Blanquet ranks (+, 1, 2, 3, 4, 5) that we replaced with their mean percentage cover (0.5%, 5%, 17.5%, 37.5%, 62.5% and 87.5%, respectively), after Tüxen and Ellenberg (1937).

### 2.3 Species occurrence data

To model the environmental niche of edelweiss, we first assembled a database with known occurrences across the Romanian Carpathians. A total of 443 presence points were amassed: 424 points that we had personally collected since 2012 and 19 additional points from published sources and local collaborators (Frink, 2015; Frink et al., 2015; Iancu & Decei, 1964; Mihăilescu, 2001; Pușcaru‐Soroceanu and Pușcaru, 1971; Resmeriță, 1973). As we later worked with predictors that had a spatial resolution of 30 arc‐seconds (ca. 1 km), more than one presence point was possible within the same raster cell. Duplicates were removed to minimize spatial autocorrelation and therefore our total number of occurrence records decreased from 424 to 47.

Given the lack of true absence data, pseudo‐absences were generated using the *biomod2* package with the *sre* strategy. This approach generates a surface range envelope model using 95% of presence data and generates pseudo‐absences outside these broadly defined environmental conditions (Barbet‐Massin et al., 2012). Ten times more absences than presences were created after Chefaoui and Lobo (2008).

### 2.4 Bioclimatic and environmental data

Initially, we prepared 25 climatic and topographic predictors (Table S2) with a gridded spatial resolution of 30 arc‐seconds (ca. 1 km). 19 bioclimatic variables were acquired from the WorldClim database, while 6 topographic variables (slope, aspect, surface roughness, Terrain Ruggedness Index, Topographic Position Index, Topographic Wetness Index) computed from WorldClim's altitude data (Hijmans et al., 2005). The importance of each variable was tested by fitting univariate generalized linear models with the variable as a predictor and species presence as the response. Goodness of fit for each model was calculated as 1—residual variance/null variance. Predictors with a variable importance <0.30 were excluded as these had relatively flat response curves and so made little difference to model predictions. To reduce multicollinearity, we further retained the predictors with the highest goodness of fit when variables were intercorrelated, as determined by a Spearman's correlation coefficient ≥0.70 (Gomes et al., 2018). Four variables were retained following this process: (i) mean temperature of the coldest quarter, (ii) precipitation of the warmest quarter, and (iii) Terrain Ruggedness Index (TRI), which is the mean of the absolute differences between the elevation of a 30 arc‐second grid cell and its 8 surrounding cells (Hijmans & van Etten, 2016). TRI values between roughly 160 to 500 m are considered intermediately/moderately rugged, with values from 500 to 960 m indicating high ruggedness (Riley et al., 1999). Although the spatial scale of the TRI measurements greatly exceeds that of our focal plants, it provides a general indicator of mountainous terrain and thus habitat where edelweiss is commonly found (Resmeriță, 1973).

We also projected future distributions under two scenarios developed by the Intergovernmental Panel on Climate Change: Representative Concentration Pathway (RCP) 4.5 and 6.0 (van Vuuren et al., 2011). We chose these scenarios because future climate conditions are likely to lie somewhere between them by the year 2100 (Hausfather & Peters, 2020). For the two scenarios, we used three Global Circulation Models (GCMs): the Met Office climate model (HadGEM2‐ES), the Model for Interdisciplinary Research on Climate Change (MIROC5) and the Norwegian Earth System Model (NorESM1‐M). These models were chosen because of their different future trends regarding our chosen predictors in the study area: HadGEM2‐ES predicts a high increase in air temperature and a moderate decrease in precipitation, MIROC5 predicts a high increase in both air temperature and precipitation, and NorESM1‐M predicts a high increase in air temperature and moderate decreases in precipitation. Rasters for future conditions in 2050 were also acquired from WorldClim and represent downscaled global projections from the 5th phase of the Coupled Model Intercomparison Project. The warmest quarter was consistent across time in the Romanian Carpathians.

### 2.5 Niche model fitting and evaluation

We estimated the environmental favorability for edelweiss across the Romanian Carpathians using our four climatic and topographic predictors within the *biomod2* framework (Thuiller et al., 2014) using R v.2.9 (R Core Team, 2015). The models were fitted using ten techniques: (i) generalized linear modeling, (ii) generalized boosting modeling, (iii) generalized additive modeling (GAM), (iv) classification tree analysis, (v) artificial neural networks, (vi) surface range envelopes, (vii) discriminant analysis, (viii) multivariate adaptive regression splines, (ix) random forests, and (x) maximum entropy. We used a repeated split‐sample procedure where 80% of the initial data were used to calibrate the models and 20% to evaluate them, repeating this process 10 times (Thuiller et al., 2009). Ten models without splitting the data were also fitted. Of the 110 resulting models, 84 were considered further as they had a true skill statistic (TSS) above 0.80, indicating a high predictive performance (Allouche et al., 2006). Given the high TSS values, all the 84 individual models had an identical weight of 0.01, so we simply averaged across their predictions to produce a single ensemble model. The ensemble also fitted the data very well with a TSS = 0.99. Variable importance was determined within the BIOMOD2 framework as one minus the correlation score between the original prediction and the prediction made with a permuted variable, and ranges between 0 (no importance) and 1 (high importance) (Thuiller et al., 2014). We used this ensemble to map the potential distribution of edelweiss under current and future (2050) conditions. Maps were output as a continuous probability of occurrence between 0 and 1.

As edelweiss can be found almost exclusively on a base‐rich substrate, we used an existing geology map for Romania (Ovejanu et al., 2011) to constrain all predicted occurrences. The substrates we considered were purely calcareous bedrocks and mixed bedrocks with limestone. We also removed unsuitable areas for edelweiss, such as forests, waterbodies, artificial surfaces, and agricultural areas using CORINE Land Cover 2018, v.20 (European Environment Agency, 2018) and airborne imagery. Finally, we retained only altitudes higher than 394 m a.s.l. because no current occurrence is officially reported at lower altitudes.

### 2.6 Statistical models of seed production

We tested how each of seed mass and number both per plot and per inflorescence varied with environmental favorability and human disturbance using generalized mixed‐effects models fitted with the *lmer* function in R (Bates et al., 2015). Environmental favorability corresponded with the predicted present‐day probability of occurrence at a 1 km^2^ scale from the ensemble species distribution model in which each plot was located. Values were logit‐transformed to be approximately normally distributed. Hiking trail distances were used as a proxy for human disturbance. We hypothesized that the relationship between edelweiss fitness and environmental favorability would also weaken as plants were closer to the hiking trails because edelweiss is more exposed to harvest irrespective of how favorable the environment is for reproduction. Therefore, we included a statistical interaction between environmental favorability and hiking trail distance in the models. We accounted for greater potential seed production in taller plants with more access to light by including the mean height of inflorescences per plot in the plot‐level analysis. In the inflorescence‐level analysis, we only modeled the first 3 individuals in a plot from which we collected seeds, so included their actual heights in the models. We also accounted for the impact of hemiparasites, cushion plants, and woody plants on seed production through competition and/or facilitation by including their abundances as additional covariates. Finally, we accounted for repeated measurements of the same plot and study site by including these factors as random effects in our models. All predictors were scaled to a mean of 0 and a standard deviation of 1 so that their effects were directly comparable. Seed mass was cube‐root transformed to reduce the skewness of the data. For models of seed number, we used a Poisson error structure and included a plot‐level random effect in the plot model and an observation‐level random effect in the inflorescence model to account for overdispersion. Estimated effects were considered statistically significant when 95% confidence intervals (CIs) excluded zero.

We used backward stepwise selection for model simplification. Models were compared using the Akaike information criterion (AIC). We sequentially removed predictors that resulted in the largest decrease in AIC, ensuring the more parsimonious models never had AIC values that were more than 2 units higher than the lowest observed value across the model set (Table S3). We stopped removing predictors when AIC values increased by more than 2 units. Main effects were only dropped after their interactions. Only the simplified model was reported in the Results.

We used the simplified statistical models to forecast seed number and mass per km^2^ given 2050 climate projections. We inputted the 2050 predictions of environmental favorability from the ensemble model into the fitted mixed‐effects models. All predictions were at the mean vegetation cover of the functional types and mean height of inflorescences, while using the present‐day trail network. This approach allowed us to isolate the direct effect of environmental change on future edelweiss persistence.

## 3 RESULTS

### 3.1 Environmental predictors of edelweiss distribution

**FIGURE 2:**
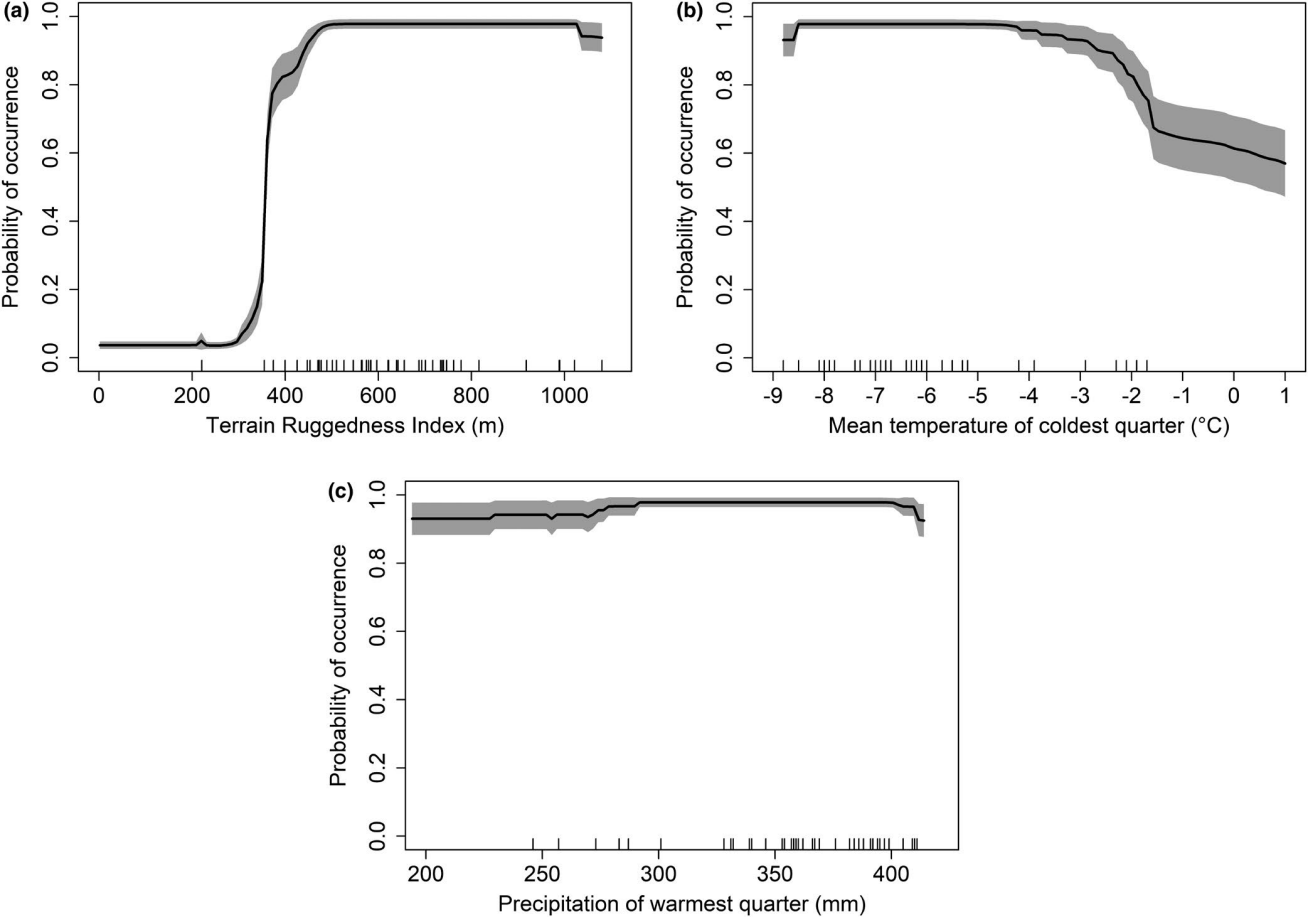
Mean response curves for *Leontopodium alpinum* for the four variables used in the species distribution models: (a) Terrain Ruggedness Index (m); (b) Mean temperature of coldest quarter (°C); (c) Precipitation of warmest quarter (mm). The distribution of observations was indicated by the vertical marks along the *x*‐axis. 95% confidence intervals are shown by the gray area

We found the edelweiss was most likely to occur in moderately and highly rugged landscapes that had cold winters and wet summers. The most important variable for the distribution of edelweiss was the Terrain Ruggedness Index (TRI), followed by the mean temperature of coldest quarter, and, to a much lesser extent, precipitation of the warmest quarter (Figure S1). Response curves from the ensemble model showed that the probability of occurrence increased strongly as landforms became moderately and highly rugged with TRI above 383 m (Figure 2a). Mean temperatures of the coldest quarter beneath −2°C, corresponding to altitudes above 130 m a. s. l. in the Romanian Carpathians, also maximized the probability of occurrence (Figure 2b). Finally, plants had a high probability of occurrence when total precipitation during the warmest quarter was above 292 mm (Figure 2c).

Future climatic conditions have the potential to reduce edelweiss’ range. Specifically, the mean temperature of coldest quarter (°C) is expected to increase under all GCMs in edelweiss’ suitable habitat in Romania (Figure S2). For example, the mean temperature of the coldest quarter is predicted to increase from −5.16 ± 0.06°C (mean ± *SE*) to −2.37 ± 0.05°C according to HadGEM2‐ES under RCP 4.5 (Figure S2). These increases will reduce the probability of occurrence for edelweiss (Figure 2a) by pushing distributions into higher and cooler elevations (Figure S3). We consequently estimated a potential net loss of between 17.2% and 35.1% of edelweiss’ habitat with a probability of occurrence >50% under future climate change (Table 1). Net loss was similar under the RCP 6.0 scenario, with 14.3 and 30.0% of grid cells currently favorable for edelweiss’ predicted to disappear (Table 1).

**TABLE 1:** Favorable habitat lost in Romania by 2050. Values are the net loss in the percentage of habitat with a probability of edelweiss occurrence >50%. Estimates were generated for three GCMs under each of two RCP scenarios

	RCP 4.5	RCP 6.0
HadGEM2‐ES	35.1	30.0
MIROC5	17.2	14.3
NorESM1‐M	24.7	16.4

Abbreviations: GCM, Global Circulation Models; RCP, Representative Concentration Pathway.

### 3.2 Current and future seed production

We found that both environmental favorability and human disturbance impacted plant fitness, though the former had relatively larger effects. As expected, seed number and seed mass both per plot and per inflorescence increased with more favorable environmental conditions (Table 2). For example, at the plot level, a 1 standard deviation (*SD*) increase in environmental favorability of 4% above the mean value of 95% increased the mean ± standard error (SE) seed number from 658.7 ± 99.8 to 994.6 ± 150.6 seeds m^−2^ and seed mass from 91.5 ± 12.9 to 142.7 ± 20.2 mg/m^2^. We also found that plants that were located far from the hiking trails had greater fitness. For example, a 1 *SD* increase in hiking trail distance of 6.9 m above the mean value of 137.9 m increased seed number from 658.7 ± 99.8 to 988 ± 149.6 seeds m^−2^ and seed mass from 91.5 ± 12.9 to 140.9 ± 19.9 mg/m.^2^

**TABLE 2:** Estimated effects and 95% confidence intervals of model predictors for present‐day seed number and seed mass both per plot and per inflorescence. Predictors retained in the simplified models included environmental favorability (Env), which was the probability of occurrence in a wider 1 km^2^ grid cell estimated by the SDMs, distance to the nearest hiking trail (Trail), and the percentage ground cover of two functional groups. Bolded values indicate statistically significant effects, that is, confidence intervals that excluded zero. We report the marginal $R_{m}^{2}$ and conditional $R_{c}^{2}$ for each model, which, respectively, consider fixed effects only and both fixed and random effects. Plot, study site, and inflorescence identity were used as random effects

Response variable	Fixed effects				$R_{m}^{2}$	$R_{c}^{2}$	Random effects
	Env	Trail	Hemiparasites	(Semi) Woody plants			
Number#/plot	**0.51**	**0.50**	**0.49**	**−0.49**	0.23	0.23	Site, plot
	**[0.02, 1.00]**	**[0.03, 0.98]**	**[0.04, 0.95]**	**[−0.97, −0.02]**			
Number#/inflorescence	**0.33**	0.23	**0.47**	**−0.56**	0.31	0.59	Plot, inflorescence, site
	**[0.05, 0.62]**	[−0.03, 0.50]	**[0.20, 0.74]**	**[−0.85, −0.29]**			
Mass/plot	**0.56**	**0.54**	**0.50**	−0.47	0.21	0.21	Site
	**[0.07, 1.04]**	**[0.06, 1.01]**	**[0.05, 0.96]**	[−0.94, <0.01]			
Mass/inflorescence	**0.25**	**0.21**	**0.35**	**−0.38**	0.31	0.61	Site, plot
	**[0.06, 0.44]**	**[0.03, 0.39]**	**[0.17, 0.54]**	**[−0.56, −0.20]**			

Other biological variables also predictably influenced plant fitness. Hemiparasites were positively associated with both seed number and seed mass both per plot and per inflorescence, while a greater coverage of (semi‐)woody plants had negative effects in all cases except on seed mass per plot (Table 2). For example, a 1 *SD* increase in the cover of hemi‐parasites of 1.3% above its mean value of 0.6% increased seed number from 92.7 ± 6.2 to 136.3 ± 9.1 seeds and seed mass from 12.5 ± 0.9 to 16.8 ± 1.2 mg per inflorescence. By contrast, a 1 *SD* increase in the cover of (semi‐)woody plants of 17.4% above its mean of 14.1% had the reverse effect, decreasing mean seed number to 40.8 ± 2.7 seeds and seed mass to 7.7 ± 0.5 mg per inflorescence. Overall, the models fitted the data moderately well, with conditional *R* ^2^ values between .21 and .61 (Table 2).

**FIGURE 3:**
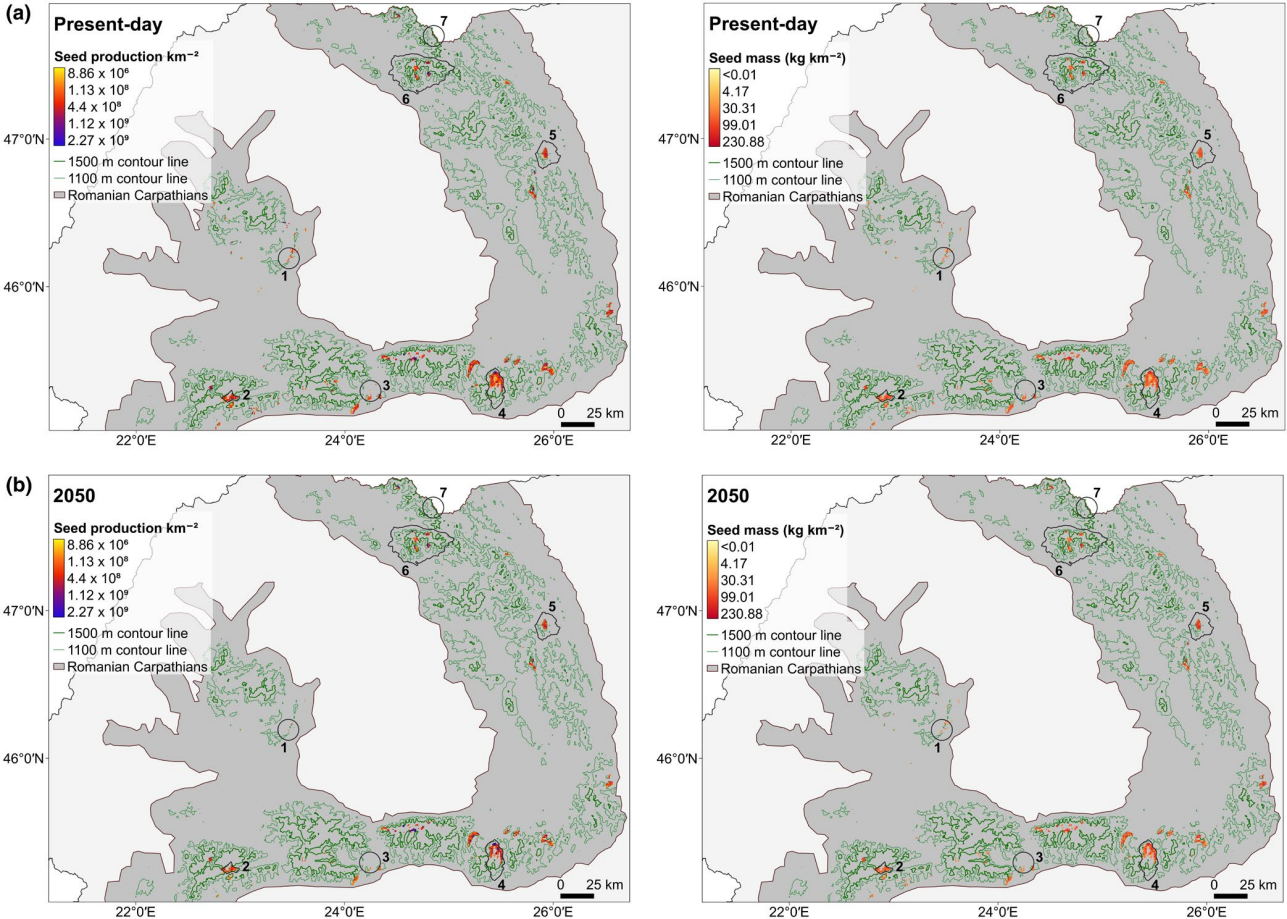
Seed number and mass for present‐day (a) and estimated in 2050 (b) using the HadGEM2‐ES, RCP 4.5 model across the suitable habitat area with a probability of edelweiss occurrence >50%. 1—Întregalde Gorges; 2—Piule‐Iorgovanu Mountains; 3—Doabra Valley; 4—Bucegi Mountains; 5—Ceahlău Mountains; 6—Rodna Mountains; 7—Coman Valley

All future scenarios estimated slight increases in the average seed number km^−2^ and seed mass km^−2^ across the habitat area with a probability of edelweiss occurrence >50% in 2050. Using HadGEM2‐ES run with RCP 4.5, the estimated average ± *SE* of seed number km^−2^ slightly increased from a mean ± *SE* of 3.5 × 10^8^ ± 6.5 × 10^6^ to 3.7 × 10^8^ ± 1 × 10^7^, while the estimated seed mass slightly increased from 65.0 ± 1.0 to 65.7 ± 1.4 kg/km^2^ (Figure 3). However, under present conditions, plants mostly occur above 456 m a.s.l, where edelweiss has a >50% probability of occurrence. As the available habitat area at higher elevations decreases with future climate change (Table 1), plants will encounter favorable conditions at altitudes over 641 m a.s.l according to HadGEM2‐ES, RCP 4.5 (Figure S3b). Therefore, seed number and seed mass will be mainly restricted to the mountains, such as Făgăraș, Bucegi or Ceahlău massifs (Figure S4), and total seed output in Romania will decline by 12 to 33% (Figure 3). These results were effectively identical with the different GCMs and RCPs (Figure S5).

## 4 DISCUSSION

By combining species distribution modeling with demographic measurements, we found that climate change may have more negative effects on alpine plant populations than human disturbances. Our results therefore support widespread evidence that alpine plant life is threatened by climate change (Dullinger et al., 2012; Engler et al., 2011; Gottfried et al., 2012) and highlight how other human activities, such as harvesting and fragmentation, can add to these impacts. One limitation of our study is that we did not consider that local microclimatic variation created by topographic heterogeneity can buffer the effects of climate change in alpine habitats (Ohler et al., 2020; Scherrer & Körner, 2011; Suggitt et al., 2018). However, macroclimate is still informative over the large spatial extent of our study, and local measurements may only improve the explained variation of our SDMs rather than change our regional predictions (Lembrechts et al., 2019). More broadly, our results showcase how demographic information can extend species distribution models beyond probabilities of occurrence to measures more directly related to long‐term population persistence, such as sexual reproduction (Dullinger et al., 2012; Fordham et al., 2013; Keith et al., 2008). Predicting seed production in the present‐day and future from SDMs, respectively, improves predictions of population dynamics and helps identify sites where plants are failing to recruit even if they are persisting. For these reasons, we can recover more information about future risks to populations than using a SDM alone. Our approach can therefore be used more generally across species and regions and complement more mechanistic demographic models that may be inapplicable in the absence of multi‐year data that are required to estimate many vital rates (Merow et al., 2014).

### 4.1 The persistence of edelweiss is impacted by climate and harvest

We found that environmental favorability and potential exposure to harvesting were individually associated with edelweiss fitness. Many studies have reported that warm temperatures and abundant rainfall correlate with seed mass and seed number by directly enhancing somatic and reproductive development (Buechling et al., 2016; Day et al., 1999; Klady et al., 2011; Kudernatsch et al., 2008; Moles et al., 2005) and indirectly increasing nutrient availability, for example, nitrogen mineralization (Smaill et al., 2011; Tanentzap et al., 2012). In edelweiss, like many other alpine plants, the growing season is too short to accumulate resources (i.e., carbohydrates and mineral nutrients) for reproduction and develop flowers (Billings & Mooney, 1968). For this reason, plants grow as rosettes for at least 1 year before flowering (Keller & Vittoz, 2015). Favorable climatic conditions would thus allow plants to accumulate more resources for reproduction, consistent with trends seen in other hapaxanthic plants whose reproductive output increases with size (Kuss et al., 2008; Metcalf et al., 2003). However, warmer future conditions contracted edelweiss’ range despite the potential benefit to fitness. Taller, shade‐casting shrubs that are favored under a warming climate can outcompete the short‐statured, light‐demanding edelweiss (Gottfried et al., 2012), consistent with the negative effect of woody plants we observed on plant fitness. This interpretation would also explain the positive effect of the Terrain Ruggedness Index, represented by high‐altitude cliffs and rocky grasslands, which can benefit fitness by providing drier conditions that limit competition for light (Ischer et al., 2014). As many studies have only forecast range shifts to predict the responses of species to climate change (Hao et al., 2019), our work suggests they may overestimate future risks where individual fitness benefits from warmer conditions. Integrating fitness estimates into range forecasts may predict more accurately the responses of species to future environmental change.

Edelweiss’ fitness also increased as plants were less accessible to harvesting, that is, further from hiking trails. This effect was remarkably strong. Only 7 m of additional distance to the average hiking trail was enough to increase both seed number and seed mass per area by nearly 50%. Others have similarly found plant fitness to be negatively impacted by trails (Chardon et al., 2018; Fenu et al., 2013). For example, the fruit density of *Silene acaulis* declined along hiking trails in Switzerland because of human trampling (Chardon et al., 2018). Few studies, however, have explicitly used the distance to hiking trails, villages, or roads as proxies for the impacts of human harvesting on plant fitness like we do here (Dhillion & Gustad, 2004; Schumann et al., 2010). The fitness impacts of harvesting will also depend on the part of the plant being removed (Ticktin & Nantel, 2004). Harvesters may selectively collect larger more apparent individuals of edelweiss that are more likely to contain seeds, subsequently reducing population regeneration. Similar effects have been purported to comprise the fitness of the American ginseng, *Panax quinquefolius* (Mooney & McGraw, 2007). Nonetheless, the weaker effect on plant fitness of harvesting than climate change may have arisen because removal of inflorescences induced the compensatory allocation of stored resources toward future reproduction (Lehtilä & Ehrlén, 2005). Experimental clipping of inflorescences while monitoring reproductive output over several seasons in relation to underground rhizome connections could help inform the design of sustainable harvesting practices that supported local communities while simultaneously promoting conservation.

### 4.2 Effects of biological variables on seed production

We found that other biological variables predictably influenced edelweiss fitness. For example, (semi‐)woody plants affect smaller herbaceous plants by shading, reducing soil moisture, and increasing soil acidity (Gabay et al., 2012; Kröpfl et al., 2002; Makarov et al., 2019), and these effects can explain their negative association with the light‐demanding edelweiss that grows mostly on base‐rich soils. Hemiparasites, none of which infect edelweiss, may have also had a facilitative effect by infecting co‐occurring species, especially belonging to the Poaceae, Rosaceae, and Fabaceae families (Bao et al., 2015; Ren et al., 2010; Suetsugu et al., 2008). For example, *Rhinanthus* spp., one of the genera identified in our plots, prefers grasses and legumes in favor of nonleguminous forbs, likely due to the high root densities of dominant species (Ameloot et al., 2005). Finally, nutrients released during the decomposition of hemi‐parasites can stimulate the primary production of host and nonhost species (Demey et al., 2013; Spasojevic & Suding, 2011), ultimately increasing seed production.

### 4.3 Conservation implications

Alpine plant life is particularly susceptible to warming temperatures (Gottfried et al., 2012; Pauli et al., 2012). Traits such as low dispersal ability, geographic isolation, small population size, and limited range size coupled with human disturbance may only exacerbate this threat and challenge conservation efforts (Rossi et al., 2009). Although edelweiss is widespread in the Romanian Carpathians, most of its populations are restricted to a few individuals, a pattern also observed in the Swiss Alps (Ischer et al., 2014). Moreover, there is already poor connectivity among populations as the distribution of basic soils in the Romanian Carpathians is fragmented. This situation may limit the exchange of seeds and pollen among populations and their long‐term persistence. Future conservation of edelweiss could focus on high‐altitude areas with large base‐rich substrates such as the Piatra Craiului Mountains. For an effective conservation strategy under present climate conditions, human harvesting should also be limited by tightening sanctions against the collection of protected plant species like edelweiss, increasing conservation awareness within local communities, and monitoring and educating tourists. However, our study also shows that the effects of present‐day disturbances, and thus their corresponding interventions, may change with future climate conditions. Conservation efforts therefore need to consider how the impacts of human disturbances may be modified by future environmental change (Solár & Janiga, 2013) and our study provides a general approach for doing so.

## CONFLICT OF INTEREST

None declared.

## AUTHOR CONTRIBUTIONS

**Lăcrămioara M. Maghiar:** Conceptualization (equal); Data curation (equal); Formal analysis (equal); Investigation (lead); Writing—original draft (equal); Writing—review and editing (equal). **Ilie A. Stoica:** Data curation (equal); Formal analysis (equal). **Andrew J. Tanentzap:** Conceptualization (equal); Data curation (equal); Formal analysis (lead); Supervision (lead); Writing—original draft (equal); Writing—review and editing (equal).

### OPEN RESEARCH BADGES

This article has earned an Open Data, for making publicly available the digitally‐shareable data necessary to reproduce the reported results. The data is available at https://doi.org/10.5061/dryad.cz8w9gj3x.

## Supporting information

Appendix S1

## Data Availability

Data available from the Dryad Digital Repository: https://doi.org/10.5061/dryad.cz8w9gj3x.
