# Supplementary material for: Integrating demography and distribution modeling for the iconic Leontopodium alpinum Colm. in the Romanian Carpathians
Source: Ecol Evol. 2021 Aug 25;11(18):12322–34. doi: 10.1002/ece3.7864 (PMC8462177; doi:10.1002/ece3.7864)
Supplement: Supplementary file 1 — Appendix S1 [file ECE3-11-12322-s001.docx]

**Appendix S1. Supplementary tables and figures**

**Table S1** Plant functional groups recorded in plots

| **Functional group** | **Plant species** |
| --- | --- |
| cushion plants | *Primula minima* |
| cushion plants | *Saxifraga aizoides* |
| cushion plants | *Saxifraga corymbosa* |
| cushion plants | *Saxifraga marginata* |
| cushion plants | *Saxifraga oppositifolia* |
| cushion plants | *Saxifraga paniculata* |
| hemiparasites | *Euphrasia salisburgensis* |
| hemiparasites | *Pedicularis* sp. |
| hemiparasites | *Pedicularis verticillata* |
| hemiparasites | *Rhinanthus* sp. |
| semi-woody plants | *Helianthemum alpestre* |
| semi-woody plants | *Helianthemum nummularium* |
| semi-woody plants | *Thymus comosus* |
| semi-woody plants | *Thymus praecox* |
| semi-woody plants | *Thymus pulcherrimus* |
| semi-woody plants | *Vaccinium vitis-idaea* |
| woody plants | *Clematis alpina* |
| woody plants | *Cotoneaster* sp. |
| woody plants | *Dryas octopetala* |
| woody plants | *Juniperus communis* |
| woody plants | *Larix decidua* |
| woody plants | *Picea abies* |
| woody plants | *Pinus mugo* |
| woody plants | *Rhododendron myrtifolium* |
| woody plants | *Rubus* sp. |
| woody plants | *Salix retusa* |
| woody plants | *Salix* sp. |

**Table S2** Climatic and topographic variables used in the modeling process

| **Category** | **Variable** | **Description** | **Units** |
| --- | --- | --- | --- |
| Climate | bio1 | Annual mean temperature | °C |
|  | bio2 | Mean diurnal range | °C |
|  | bio3 | Isothermality | % |
|  | bio4 | Temperature seasonality | °C |
|  | bio5 | Maximum temperature of warmest month | °C |
|  | bio6 | Minimum temperature of coldest month | °C |
|  | bio7 | Temperature annual range | °C |
|  | bio8 | Mean temperature of wettest quarter | °C |
|  | bio9 | Mean temperature of driest quarter | °C |
|  | bio10 | Mean temperature of warmest quarter | °C |
|  | bio11 | Mean temperature of coldest quarter | °C |
|  | bio12 | Annual precipitation | mm |
|  | bio13 | Precipitation of wettest month | mm |
|  | bio14 | Precipitation of driest month | mm |
|  | bio15 | Precipitation seasonality | % |
|  | bio16 | Precipitation of wettest quarter | mm |
|  | bio17 | Precipitation of driest quarter | mm |
|  | bio18 | Precipitation of warmest quarter | mm |
|  | bio19 | Precipitation of coldest quarter | mm |
| Topography | slp | Slope | ° |
|  | asp | Aspect | ° |
|  | rough | Surface roughness | m |
|  | TRI | Terrain Ruggedness Index | m |
|  | TPI | Topographic Position Index | n/a |
|  | TWI | Topographic Wetness Index | n/a |

**Table S3** AIC values of models fitted to seed number and mass either per plot or inflorescence. Bolded models

indicate those reported in the Main Text. Predictors included the probability of occurrence in a wider 1km^2^

grid cell estimated by the species distribution models (env), distance to the nearest hiking trail (trail), plant

height (height), and the percentage ground cover of cushion (cushion), hemiparasite (hemip), and (semi-)woody

(woody) plants in the corresponding plot. All plot level models included study site as a random effect, whereas

inflorescence models also included plot. To account for overdispersion, all seed number per inflorescence

models also included an observation-level random effect.

| **Model** | **AIC** |
| --- | --- |
| **number/plot** |  |
| trail × env + trail + env + cushion + height + hemip + woody | 688.45 |
| trail + env + cushion + height + hemip + woody | 686.53 |
| trail + env + cushion + hemip + woody | 684.97 |
| **trail + env + hemip + woody** | **686.97** |
| **number/inflorescence** |  |
| trail × env + trail + env + cushion + height + hemip + woody | 1303.28 |
| trail + env + cushion + height + hemip + woody | 1301.51 |
| trail + env + cushion + hemip + woody | 1300.32 |
| **trail + env + hemip + woody** | **1300.80** |
| **mass/plot** |  |
| trail × env + trail + env + cushion + height + hemip + woody | 188.13 |
| trail + env + cushion + height + hemip + woody | 186.01 |
| trail + env + height + hemip + woody | 183.61 |
| **trail + env + hemip + woody** | **182.60** |
| **mass/inflorescence** |  |
| trail × env + trail + env + cushion + height + hemip + woody | 264.91 |
| trail + env + cushion + height + hemip + woody | 261.40 |
| trail + env + height + hemip + woody | 256.55 |
| **trail + env + hemip + woody** | **256.15** |

**
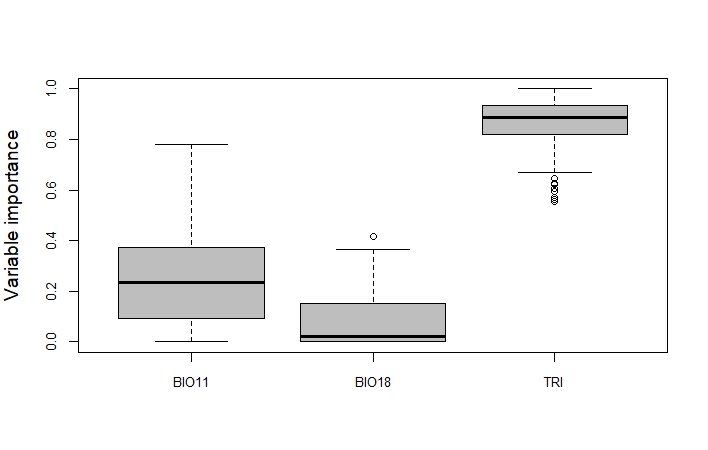
**

**Fig. S1.** Importance of each predictor used in the 84 models. BIO11 = mean temperature of coldest quarter (°C);

BIO18 = precipitation of warmest quarter (mm); TRI = Terrain Ruggedness Index (m).


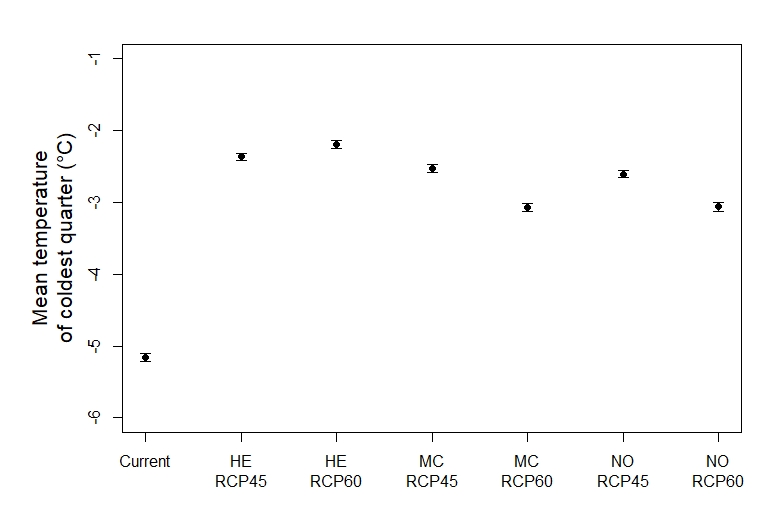


A)


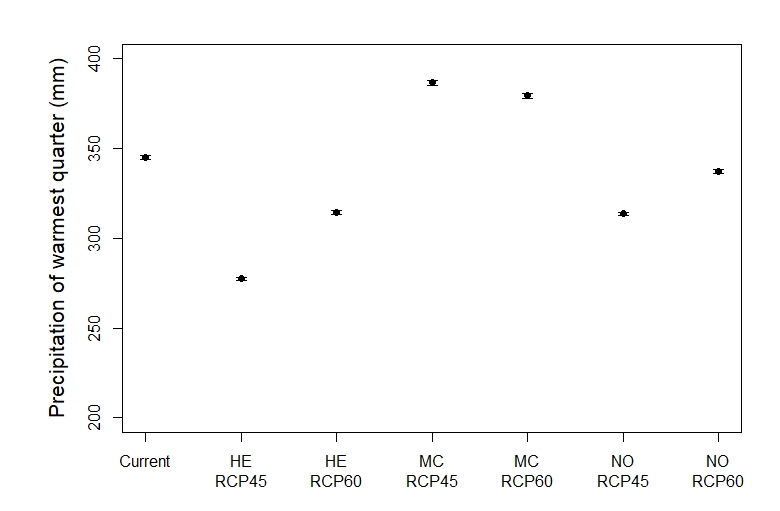


B)

**Fig. S2.** Changes in the averages of climate variables according to the three GCMs projected under RCP4.5 and RCP6.0 in the suitable habitat of edelweiss from the Romanian Carpathians. Error bars represent standard errors.

(A) Mean temperature of coldest quarter (°C); (B) Precipitation of warmest quarter (mm). HE - HadGEM2-ES; MC - MIROC5; NO - NorESM1-M.


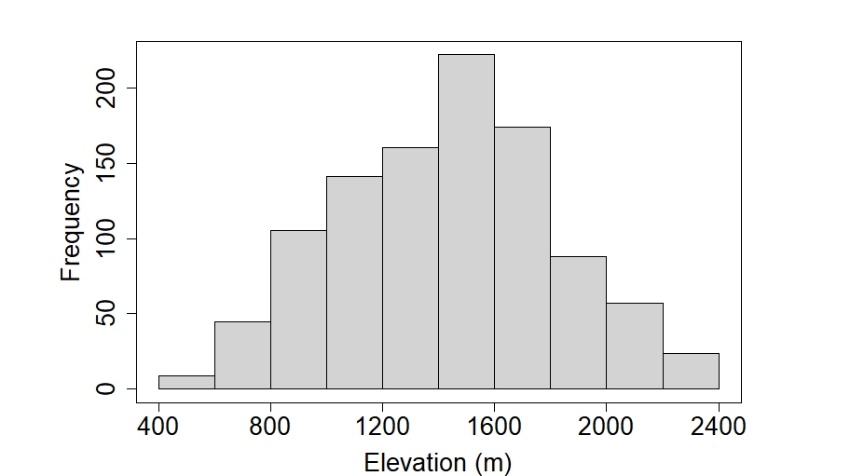


A)

**
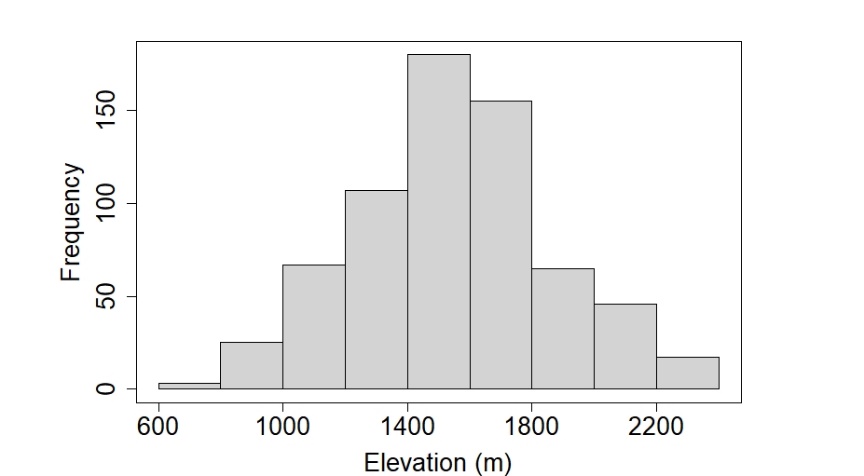

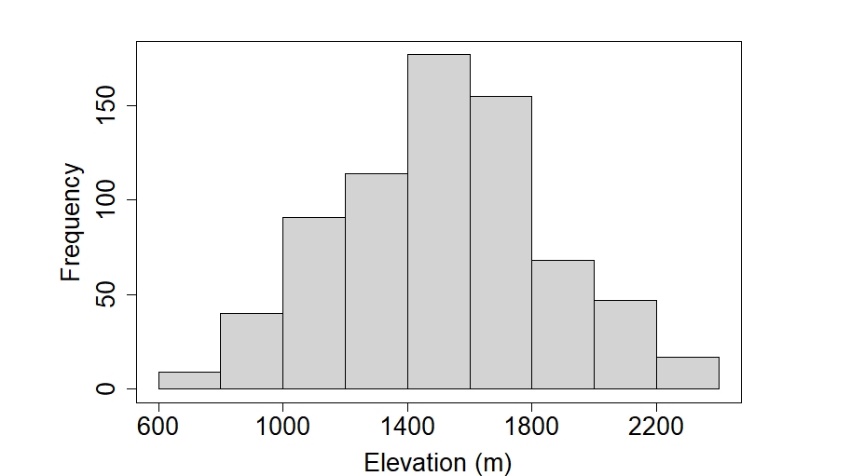
**

C)

B)

**
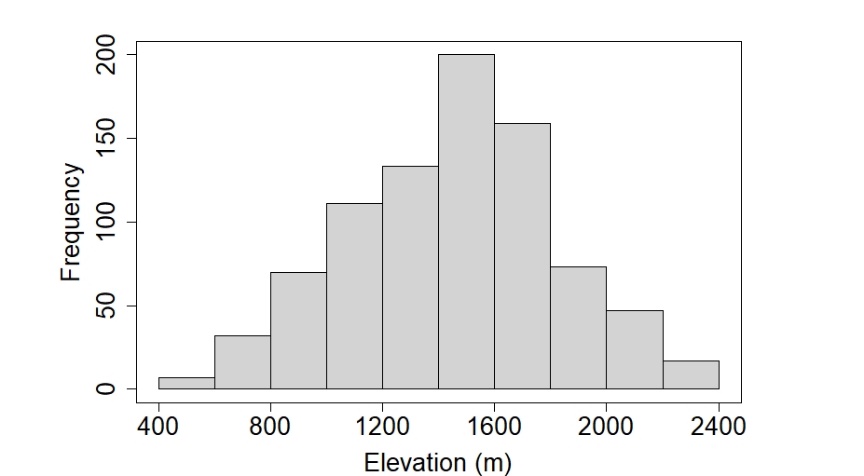

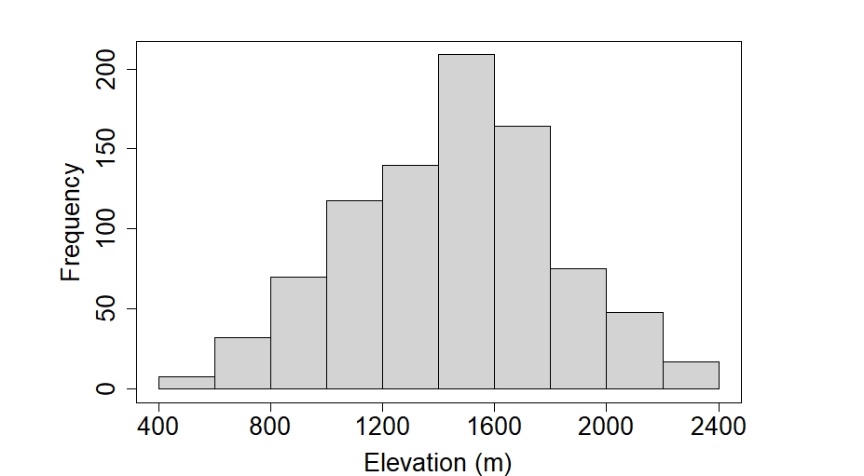
**

E)

D)

**
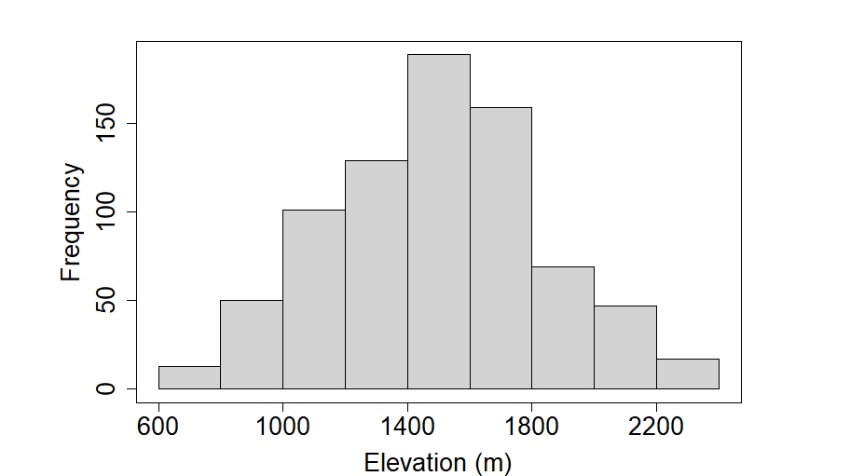

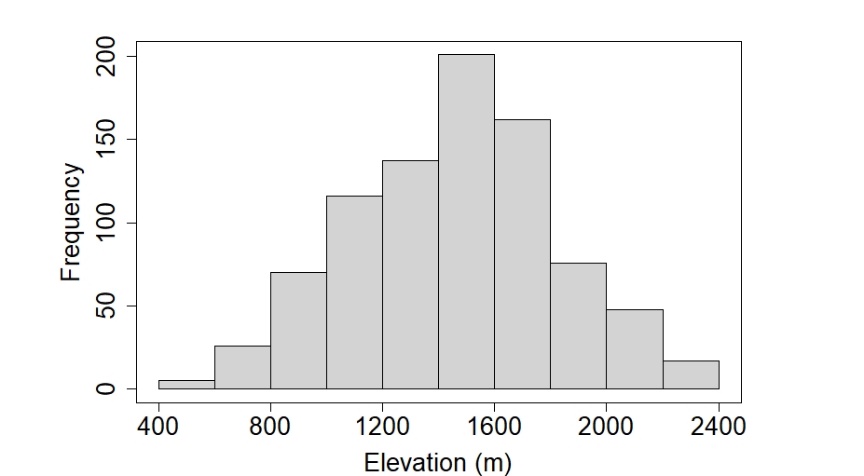
**

G)

F)

**Fig. S3.** Histograms of altitudes across the suitable habitat area with a probability of edelweiss occurrence >50%. (A) Present-day; (B) HadGEM2-ES, RCP 4.5; (C) HadGEM2-ES, RCP 6.0; (D) MIROC5, RCP 4.5; (E) MIROC5, RCP 6.0; (F) NorESM1-M, RCP 4.5; (G) NorESM1-M, RCP 6.0.


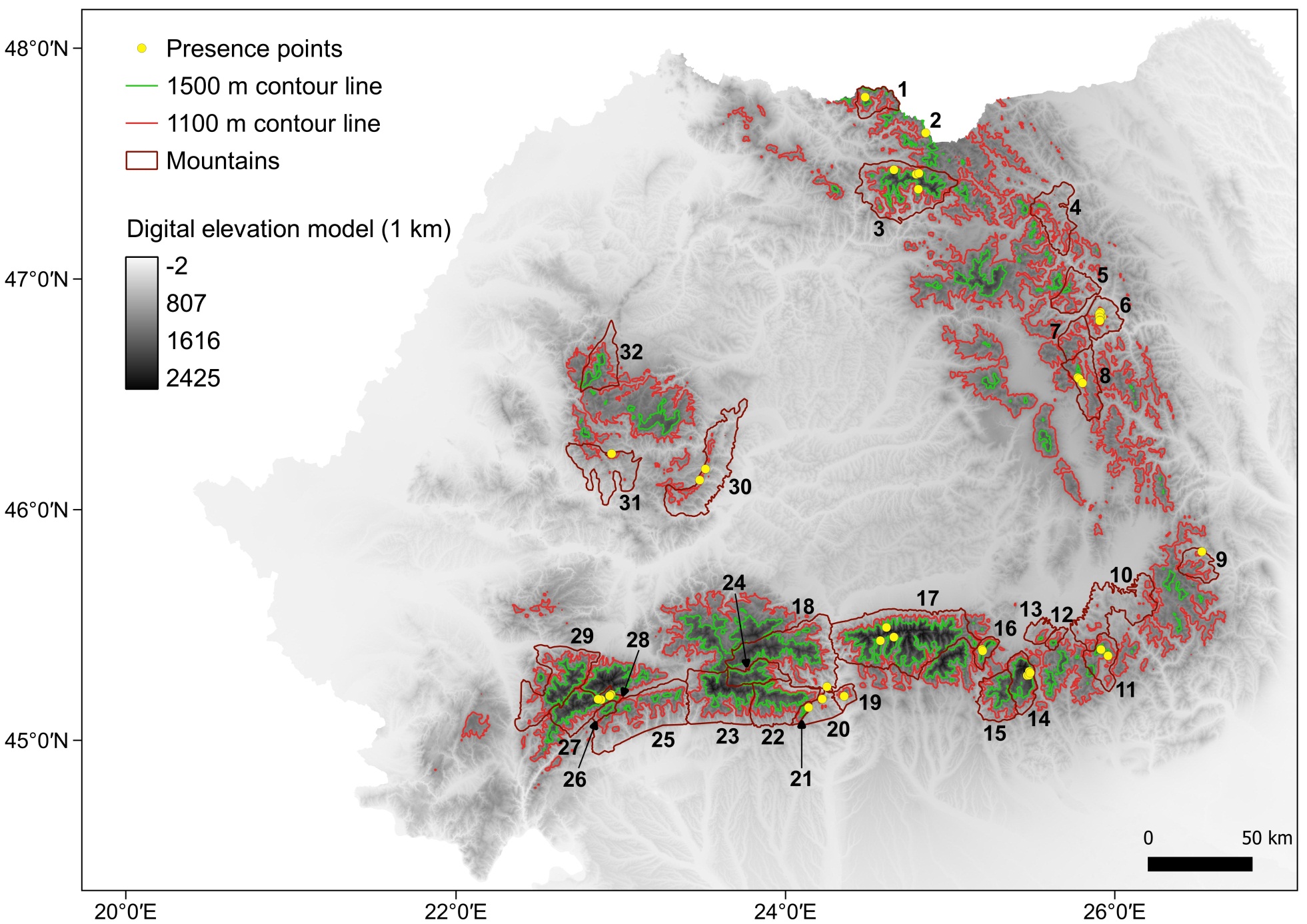


**Fig. A4.** Edelweiss’ presence points used in the SDMs.

1. Farcău Mountains, 2. Coman Valley, 3. Rodna Mountains, 4. Rarău Massif, 5. Grințieșul Mare Massif, 6. Ceahlău Massif, 7. Licaș Mountains, 8. Hășmaș Massif, 9. Coza Mountain, 10. Clăbucetele Întorsurii, 11. Ciucaș Massif, 12. Piatra Mare Massif, 13. Postăvarul Massif, 14. Bucegi Massif, 15. Leaota Mountains, 16. Piatra Craiului Massif, 17.Făgăraș Massif, 18. Lotrului Mountains, 19. Cozia Massif, 20. Sturului Mountains, 21. Buila-Vânturariţa Massif, 22. Căpățânii Mountains, 23. Parâng Massif, 24. Latoriței Mountains, 25. Vâlcan Mountains, 26. Oslea Massif, 27. Godeanu Massif, 28. Piule-Iorgovanu Mountains, 29. Țarcu Massif, 30. Trascău Mountains, 31. Găina Mountain, 32. Vlădeasa Ma


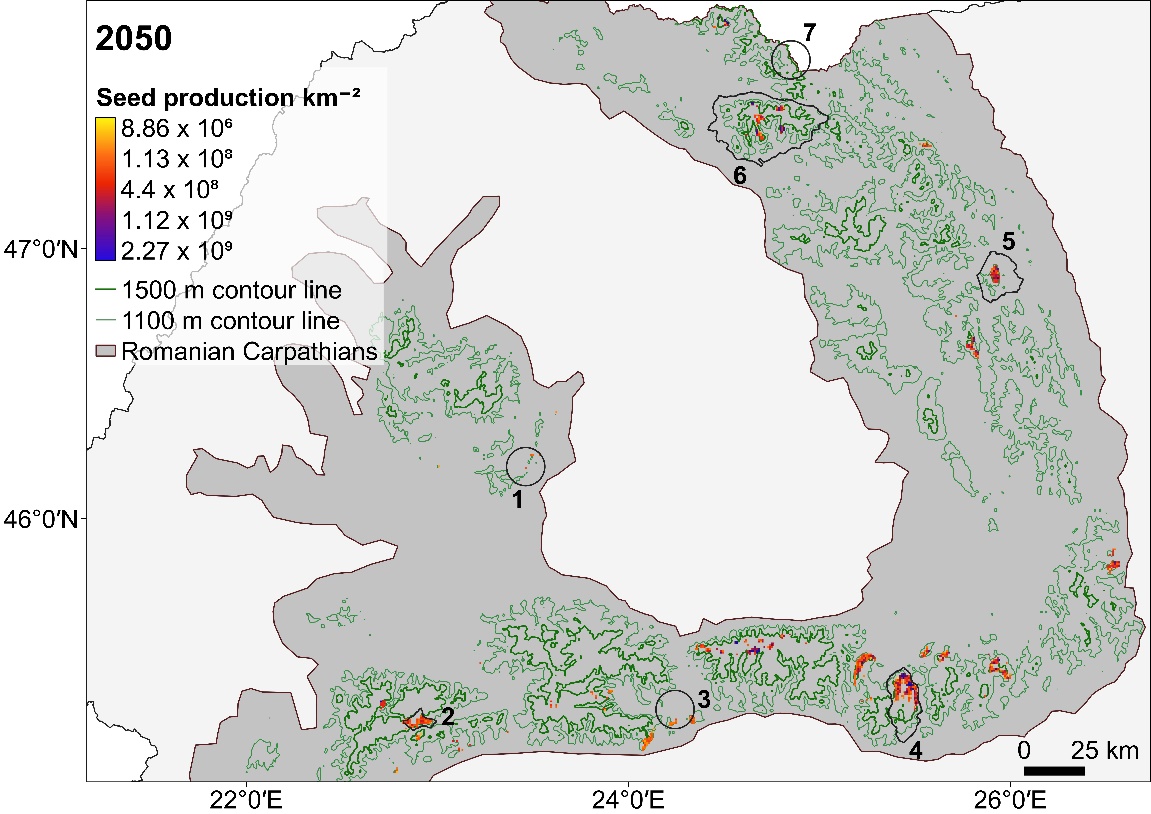

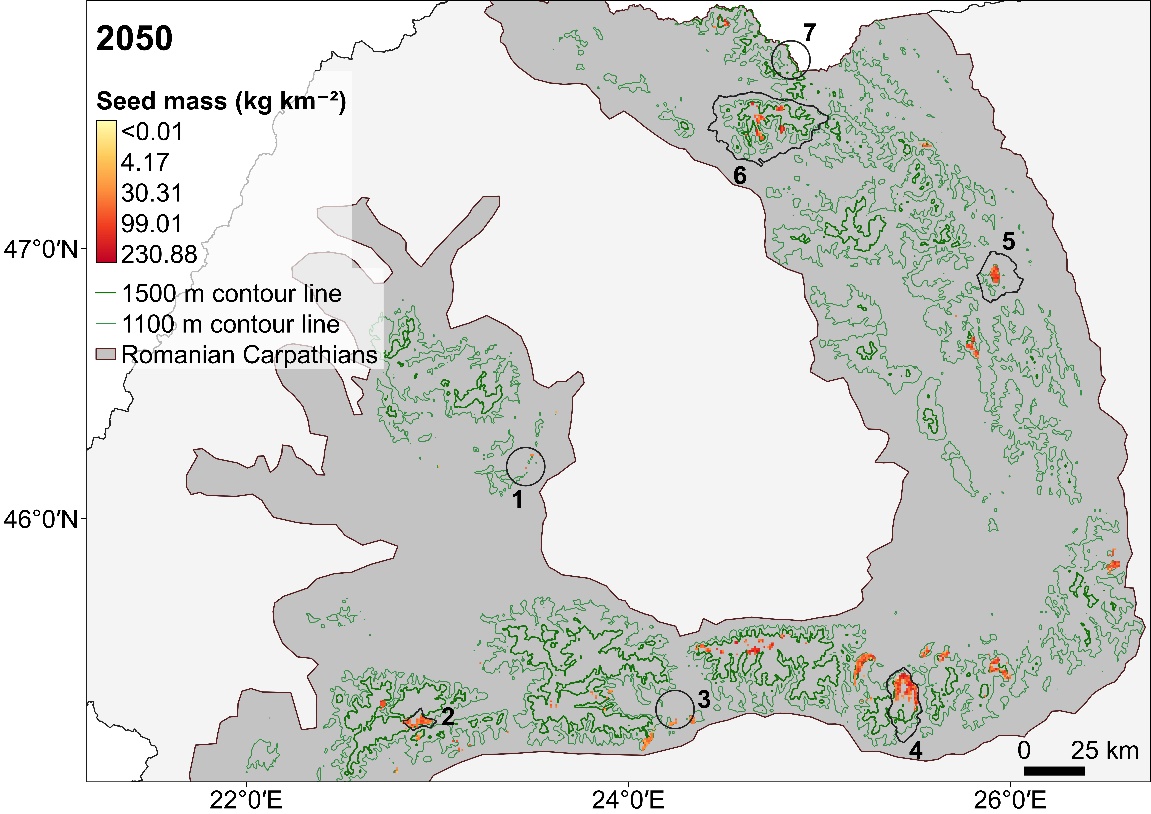


**A**


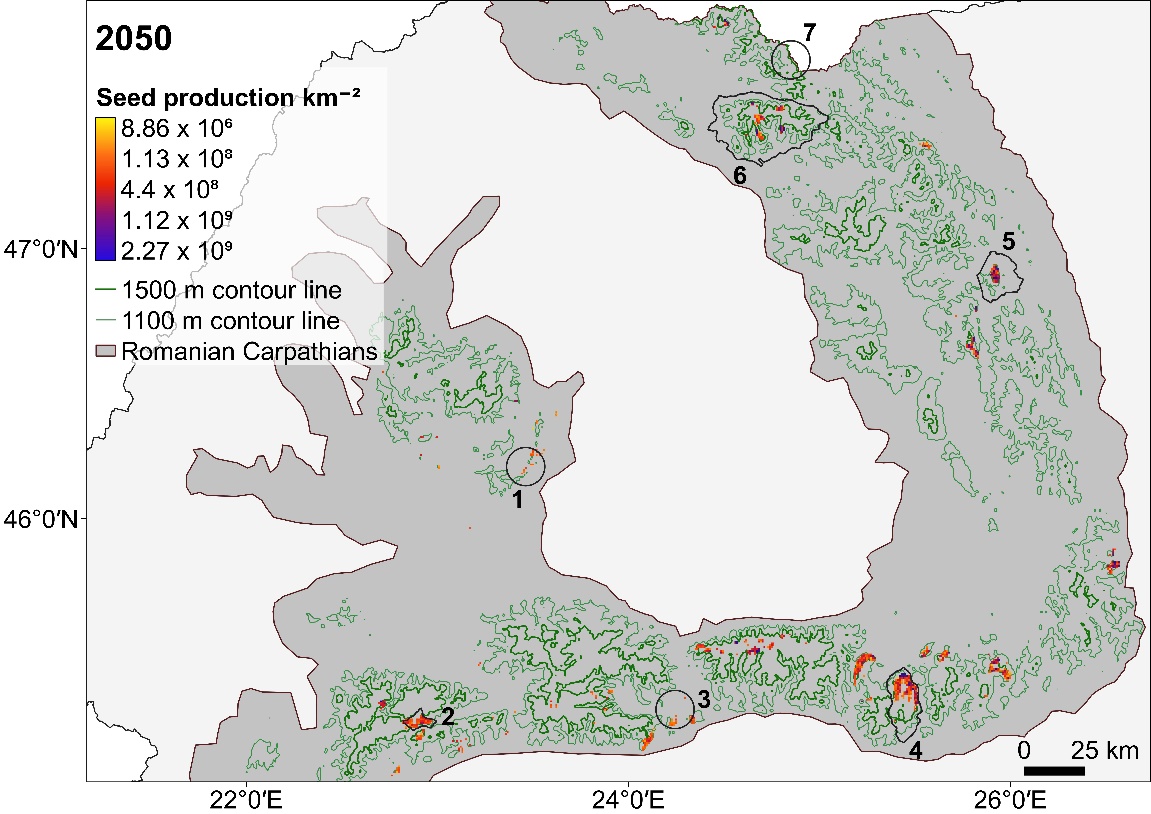

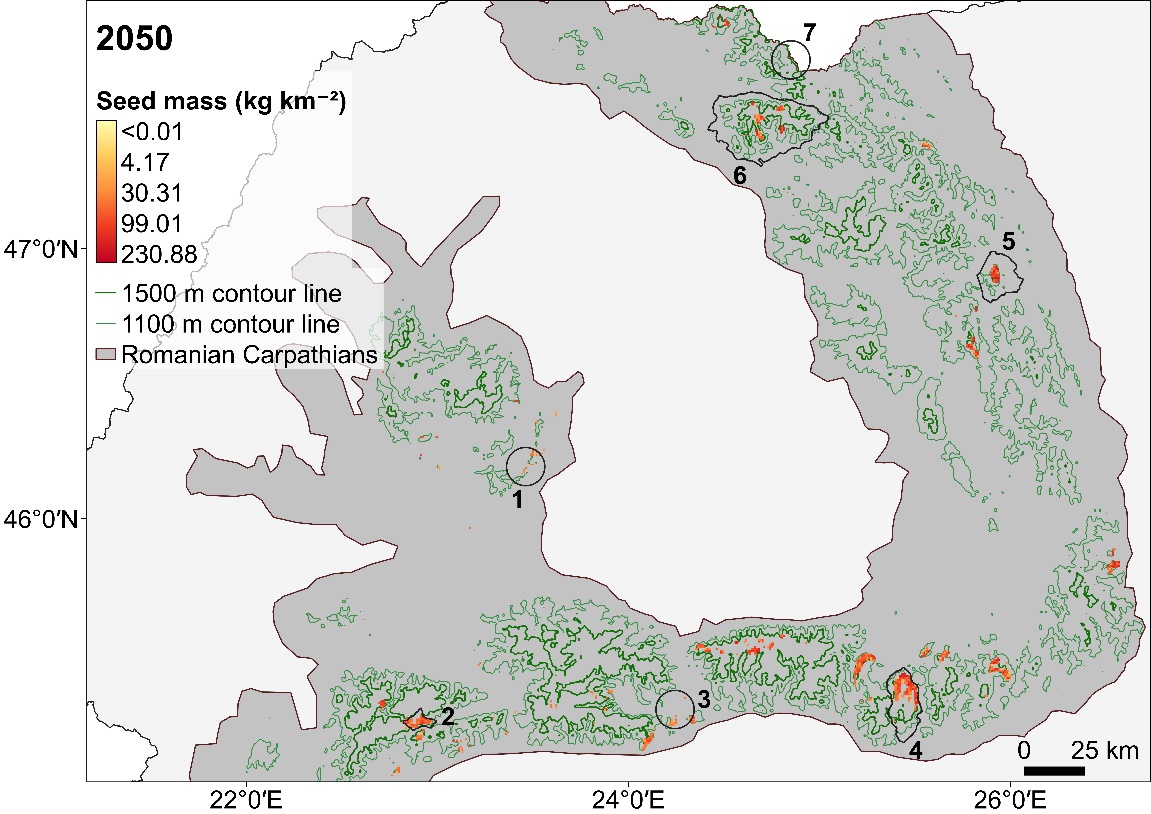


**B**


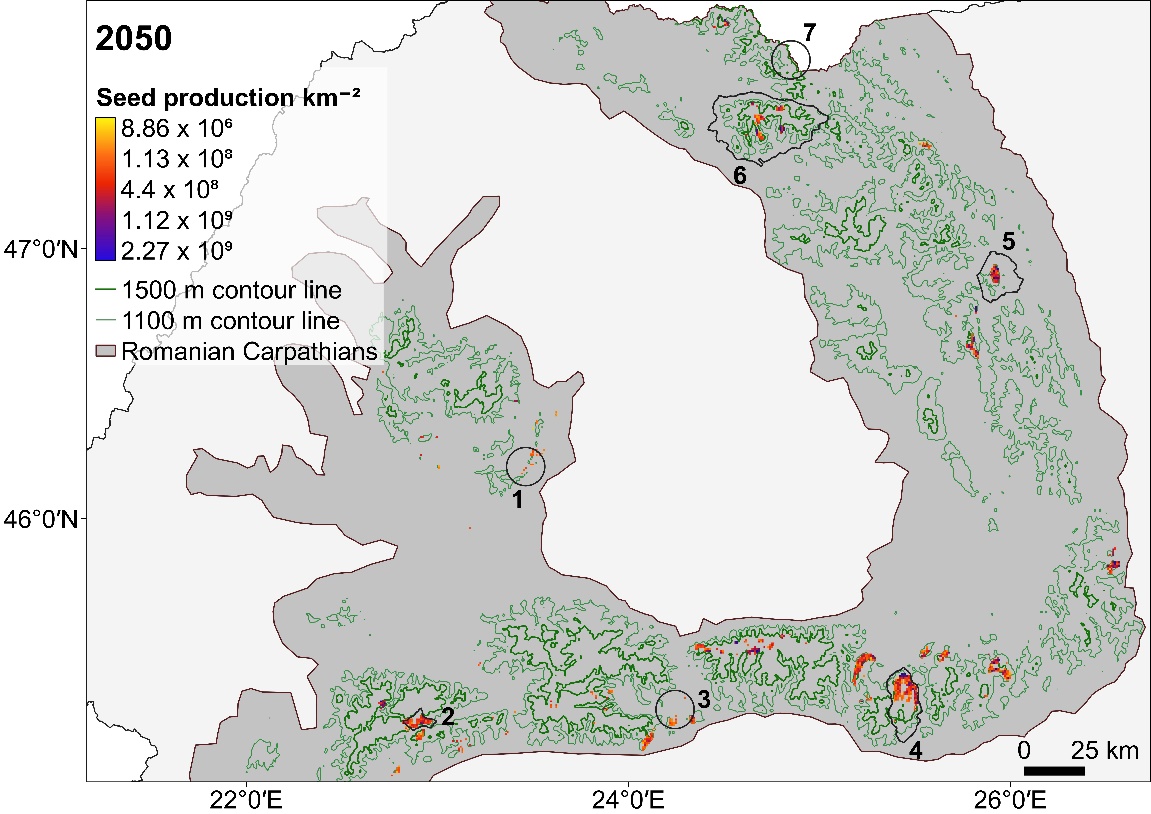

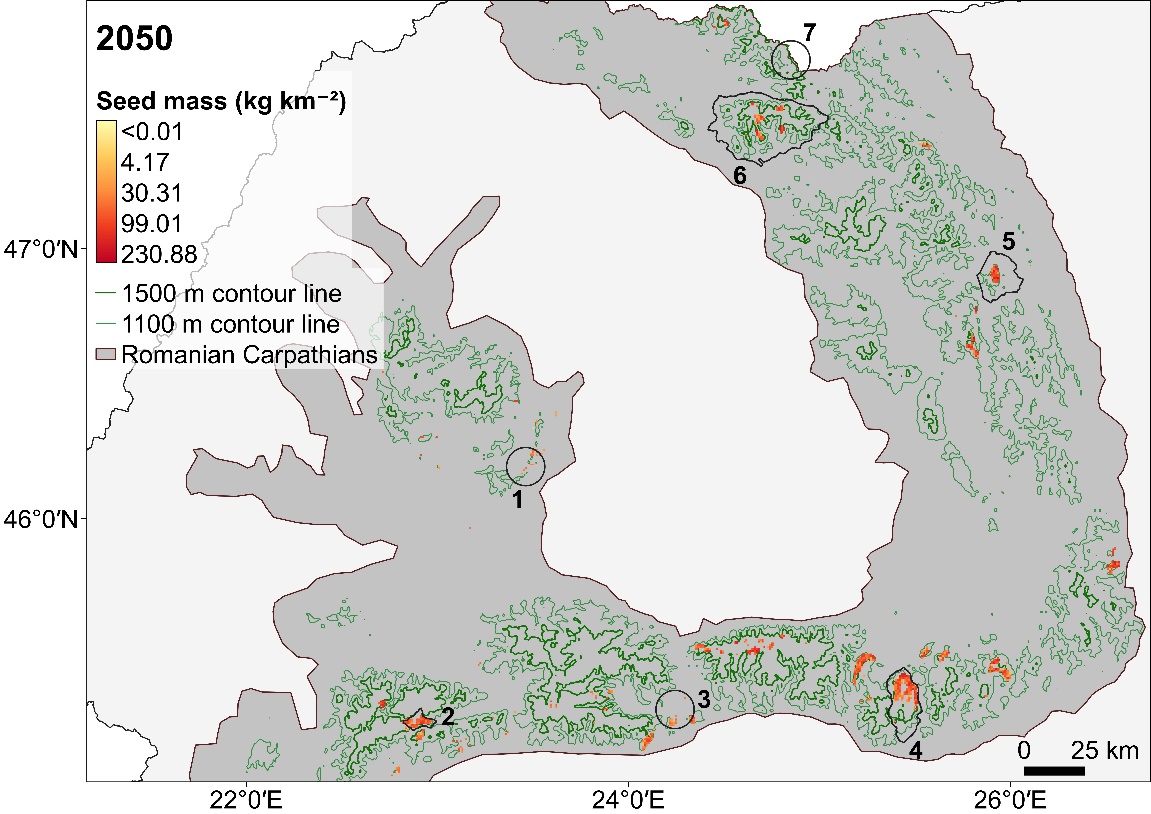


**C**


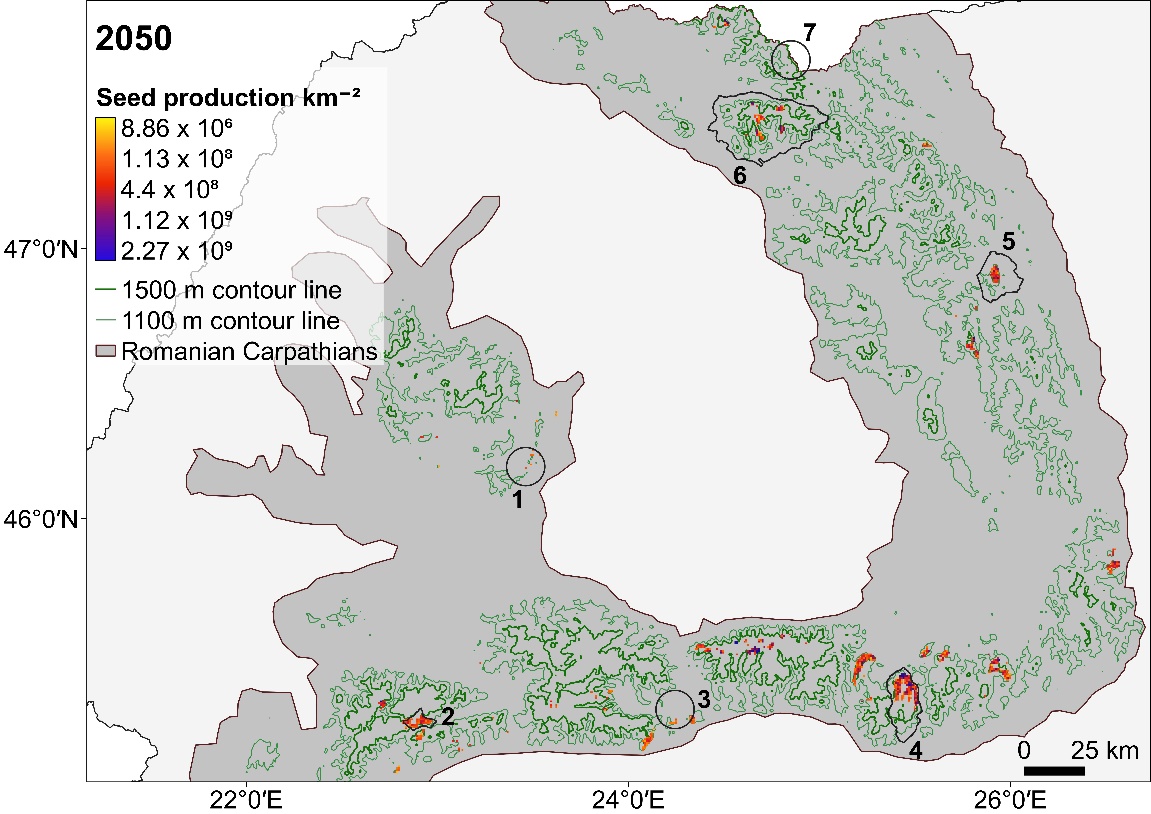

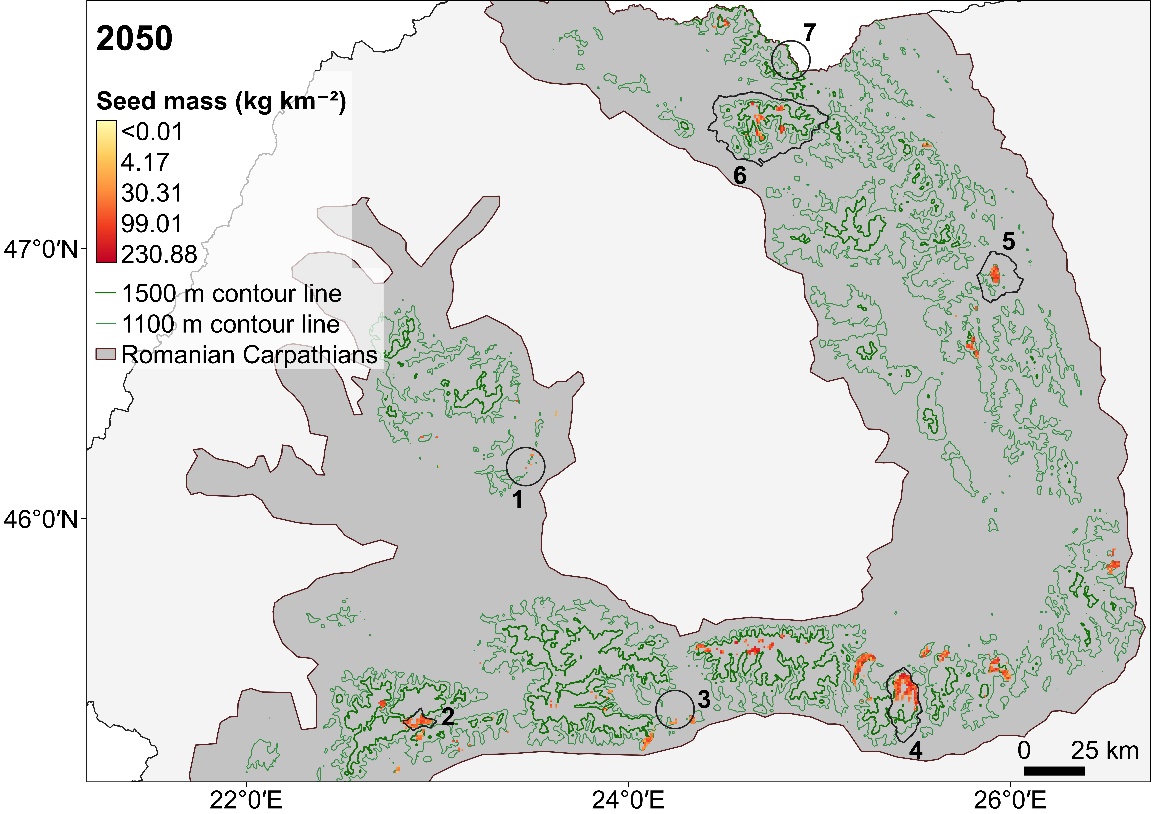


**D**


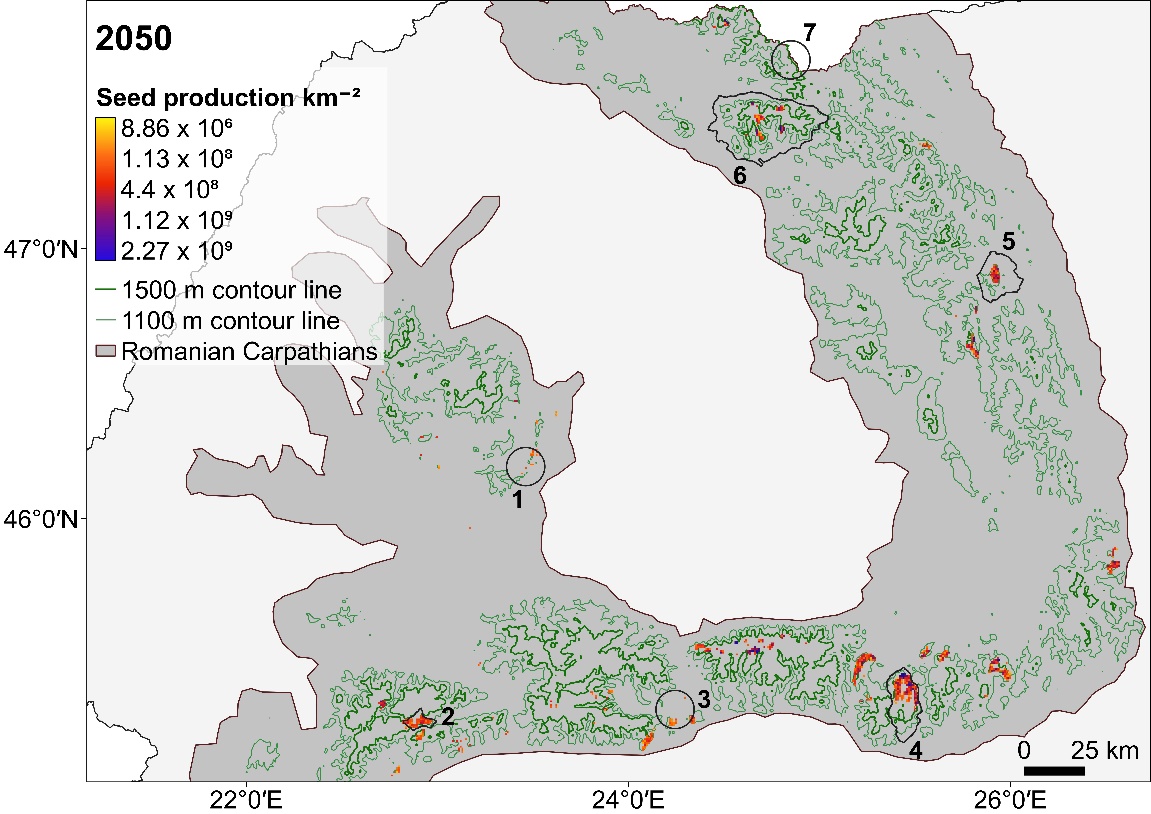

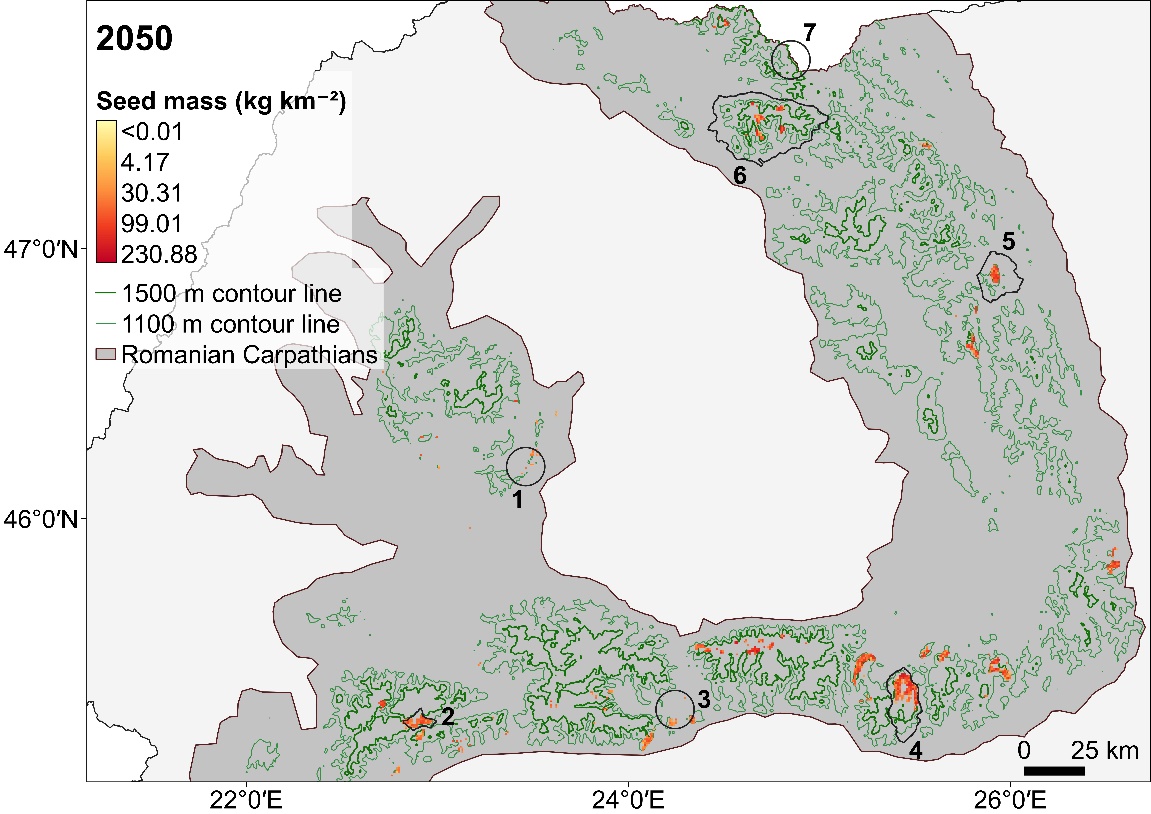


**E**

**Fig. S5.** Seed production and mass estimated in 2050 across the suitable habitat area with a probability of edelweiss occurrence >50%

using the following models: (A) HadGEM2-ES, RCP 6.0; (B) MIROC5, RCP 4.5; (C) MIROC5, RCP 6; (D) NorESM1-M, RCP 4.5; (E) NorESM1-M, RCP 6.0.

1 – Întregalde Gorges; 2 – Piule-Iorgovanu Mountains; 3 – Doabra Valley; 4 – Bucegi Mountains; 5- Ceahlău Mountains; 6 – Rodna Mountains; 7 – Coman Valley*.*
